# Caloric content and industrial processing modulate the mental simulation of food concepts

**DOI:** 10.1007/s00426-026-02379-2

**Published:** 2026-09-11

**Authors:** Sofia Gentili, Enrico Collantoni, Valentina Meregalli

**Affiliations:** 1 Department of Neuroscience, University of Padua, Padova, Italy; 2 Padua Neuroscience Center, University of Padua, Padova, Italy

**Keywords:** Grounded cognition, Mental simulation, Ultra-processed foods, Sensorimotor norms, Food reward, Eating behavior

## Abstract

**Introduction:**

Grounded cognition theories propose that concepts, including food stimuli, are represented through the partial reactivation of sensory and motor experiences. Evidence suggests that foods subjectively perceived as unhealthy tend to elicit stronger sensorimotor simulations, which may, in turn, increase their perceived attractiveness and consumption. However, it remains unclear to what extent objectively defined food properties, specifically caloric content and degree of industrial processing, modulate this simulation.

**Methods:**

In this study, 99 Italian-speaking participants rated 50 food words (25 highly processed; 25 minimally processed) across six sensory modalities (interoception, touch, hearing, olfaction, taste, vision), a motor dimension, and measures of wanting and liking. Food categories were matched for caloric content to isolate the effect of processing level.

**Results:**

Linear mixed-effects models revealed a significant and consistent interaction between caloric content and degree of processing in modulating mental simulation across all sensory modalities, the global perceptual index (Minkowski3), and motor simulation. Specifically, higher caloric content was associated with stronger sensorimotor simulation only for highly processed foods, whereas this relationship was not significant for minimally processed ones. Furthermore, higher levels of simulation, both perceptual and motor, predicted greater wanting and liking, independently of the objective properties of the foods.

**Conclusions:**

These findings indicate that industrial processing constitutes a relevant variable, distinct from caloric content alone, in shaping sensorimotor food representations, and suggest a possible cognitive mechanism through which ultra-processed foods may acquire heightened motivational salience, with potential implications for understanding dysregulated eating behavior and designing interventions to promote healthier food choices.

**Supplementary Information:**

The online version contains supplementary material available at https://doi.org/10.1007/s00426-026-02379-2.

## Introduction

Eating behavior extends well beyond a simple biological imperative. It encompasses not only the act of consumption, but also the broader set of selection processes through which individuals navigate their nutritional environment. In contemporary societies, these behaviors extend beyond the fulfillment of basic nutritional and metabolic needs. Instead, they are shaped by a confluence of psychological, social and commercial factors (e.g. emotional states, social context, habits, marketing advertising, affordability and fast-paced lifestyles) alongside the widespread availability and optimized properties of modern food products (Chen & Antonelli, 2020; Jayasinghe et al., 2025). In this context, ultra-processed foods (UPFs) play a central role in current dietary choices due to their convenience, long shelf life, and hyper-palatable properties, often being preferred over whole-foods despite their poorer nutritional profile (Lane et al., 2024; Monteiro & Astrup, 2022). The consequences of UPFs consumption can be significant, increasing the risk of obesity, metabolic disorders (e.g., diabetes) and cardiovascular diseases (Ahmed et al., 2024; Juul et al., 2025) as well as potentially contributing to eating disorders characterized by loss of control, such as bulimia nervosa and binge-eating disorder (Figueiredo et al., 2022; Wiss & LaFata, 2024).

Building on these public health issues, recent research has focused on the neural and cognitive mechanisms underlying food representation and selection. Understanding how the brain represents food and its associated characteristics – that is, how food is mentally conceptualized – is crucial to explain consumption choices and to inform public health strategies. Theories of grounded (or embodied) cognition provide a powerful framework for this investigation. While alternative accounts of conceptual representation exist – including classical perspectives that describe concepts as abstract, static, and hierarchically organized (e.g. the classical theory, semantic network models, and prototype theory) (Collins & Quillian, 1969; Hull, 1920; Rosch & Mervis, 1975) – grounded cognition conceives of concepts as concrete, dynamic, and adaptive, positing an inextricable link between perception, action, and cognition. This perspective suggests that human experience is fundamentally grounded in the body, arising from the dynamic interplay between sensory and motor systems and higher-order associative processes (L. W. Barsalou, 1999; Craighero, 2022; Gallese † & Lakoff, 2005). From this perspective, cognition is built upon body-dependent representations formed during sensory experience, which in turn underlie goal-directed behaviors. Central to this framework is the concept of mental simulation, defined as the partial reactivation of multimodal representation formed during direct experience and stored in memory (L. W. Barsalou, 2008).

Converging evidence from neuroimaging, electrophysiological, and behavioral studies indicates that concept representations are stored in sensory and motor systems engaged during information acquisition (Martin, 2007). Accordingly, action-related words activate the same motor, premotor, and fronto-parietal regions involved in action execution, and facilitate lexical decisions for body–object interaction words (Hauk et al., 2004; Siakaluk et al., 2008; Tettamanti et al., 2005). Importantly, these patterns extend to other types of verbal cues, as food-related ones (Goldberg et al., 2006; Kiefer et al., 2008; Moscoso del Prado et al., 2006). Passive reading of food words - particularly those associated with odour, taste or craving - mainly activates the same neural network that is involved during actual food consumption (Barrós-Loscertales et al., 2012; González et al., 2006; Pelchat et al., 2004). This network encompasses primary sensory and taste perception regions together with reward-related areas and dorsal regions supporting cognitive control (Chen et al., 2016). According to grounded cognition accounts, the reactivation of past eating experiences propagates from sensory areas into these reward and control regions, generating predictive representations that may contribute to subsequent food evaluation and choice (L. Barsalou & Papies, 2015). Importantly, not all foods trigger sensorimotor activation to the same extent: research using food pictures shows that highly palatable/highly caloric foods elicit stronger activation of sensory and reward cortices than low-calorie or bland foods (Chen et al., 2016; van der Laan et al., 2011). Likewise, UPFs may be expected to evoke strong cortical responses because of their greater pleasantness and their more intense and complex sensory properties relative to unprocessed or minimally processed foods (Lemos et al., 2022). Such amplified and multifaceted simulation could, in turn, more comprehensively reactivate the strong rewarding sensations associated with their consumption, potentially increasing the motivation to eat and leading to greater subsequent consumption (Chen et al., 2016). While these findings suggest that calorie content and processing level may influence the neural responses elicited by food cues, it remains unclear whether similar effects extend to the subjective sensorimotor simulations evoked by food concepts. Instead, most research has primarily examined sensorimotor simulation as a function of subjective perceptions of healthiness and palatability. For example, studies employing verbal cues have shown that tempting foods are described with greater richness and detail, perceived as more attractive and more strongly represented in terms of actual eating experiences (Papies, 2013). Additionally, stronger sensory simulation appears to mediate the relationship between perceived unhealthiness and attractiveness (Speed et al., 2023), consistent with the grounded cognition theory of desire and the “unhealthy-tasty” intuition (Papies et al., 2017, 2020; Raghunathan et al., 2006). Although informative, this focus has key limitations: subjective evaluations of foods (e.g., perceived temptingness, attractiveness, or healthiness) may reflect not only characteristics of the food itself but also individual interpretations, cultural norms, transient states (e.g., dietary goals), and personal beliefs. To address this gap, the present study aims to investigate how objectively defined food properties, specifically caloric content and degree of industrial processing, relate to sensorimotor simulation and to key components of food reward (i.e., wanting and liking). Building on the grounded cognition theory(Papies et al., 2020) it was hypothesized that (H1) foods higher in calories and degree of processing would elicit stronger sensory and action simulations across different modalities, and that (H2) these foods would show stronger associations with a global perceptual index of mental simulation (Minkowski3), a measure that captures overall perceptual strength (Lynott et al., 2020). Furthermore, it was hypothesized that (H3) stronger sensorimotor mental simulation would predict higher ratings of wanting and liking.

To investigate this, the methodological framework of sensorimotor norms was used. In 2020, Lynott and colleagues developed the Lancaster Sensorimotor Norms, a large-scale database providing multidimensional ratings of perceptual and motor strength for more than 40,000 English words. By asking participants to rate the strength of experience associated with a word across multiple dimensions (e.g. taste, smell, touch, vision, hearing, sensation in the body, action with different effectors), the authors derived a profile that approximates the underlying perceptual simulation evoked by each concept. Although these ratings are based on introspective judgments, they have been shown to reliably predict performance in lexical decision and word naming tasks (Connell & Lynott, 2012), tasks in which conceptual processing is known to be influenced by the activation of sensorimotor representations, thus supporting the use of perceptual strength ratings as a proxy for the strength of sensorimotor simulation evoked by a concept. Adapting the rating-based methodology used in the Lancaster Sensorimotor Norms to the food domain, we developed an Italian-language survey using food words systematically classified by degree of industrial processing and caloric content, allowing us to test whether objectively defined food properties modulate the strength and multidimensionality of sensorimotor simulation and, in turn, key components of food reward.

## Methods

### Participants

A total of 340 Italian-speaking participants initiated the online survey. Participants were recruited through online advertisements shared on social media platforms and a snowball sampling procedure. Inclusion criteria were: (1) age ≥ 18 years and (2) native speaker or excellent comprehension of the Italian language. Only participants who completed the questionnaire were retained for analysis, resulting in a final sample of 99 participants. A sensitivity analysis (200 simulations, α = 0.05) indicated that the current design (99 participants, 50 food words) had 80% power to detect interaction effects corresponding to a standardized β = 0.09, which represents a small effect size. The study was approved by the Ethics Committee of the Department of Psychology at the University of Padova (n° 1045-a) and conducted in accordance with the latest version of the Declaration of Helsinki. All participants provided written informed consent prior to participation.

### Procedure

The survey was developed and distributed online using Qualtrics XM (Qualtrics, 2025). The survey took approximately one hour to complete. At the beginning of the survey, participants provided demographic and clinical information, including age, gender, height, weight, education level, and nationality. Subsequently, they completed the sensorimotor questionnaire, described in detail in the next section.

### Sensorimotor questionnaire

For each word, participants rated the extent to which the concept elicited sensory, motor, and reward-related experiences, as well as familiarity. All ratings were provided on a 6-point Likert scale ranging from 0 (*not at all*) to 5 (*very much*).

#### Stimuli

The stimulus set comprised 75 words, each presented in randomized order: 25 highly processed food (HPF), 25 minimally processed food (MPF), and 25 manipulable objects with a clear action affordance. As object-related words were not included in the analyses of the present study, the focus from this point onward will be exclusively on the 50 food-related words. The stimulus set was purposely constructed for the present study. An initial pool of 75 HPF and 75 MPF was generated by the researchers by consensus, selecting foods commonly found in Italian cuisine, aiming to include a variety of food types and to span a broad range of caloric contents. Food items were categorized as HPF or MPF according to the NOVA Food Classification System (Monteiro et al., 2010), which classifies foods into four groups based on the extent and purpose of industrial processing. For the purposes of the present study, Groups 1 and 2 (unprocessed or minimally processed foods and culinary ingredients derived from natural sources) were combined into MPF category, whereas Groups 3 and 4 (processed and ultra-processed foods) were combined into HPF category. Caloric density (kcal/100 g) for each food item was obtained from nutritional tables provided by the Council for Agricultural Research and Economics (CREA, 2023). After calorie assignment, the initial pool was reduced to 25 items per category, selecting items so as to minimize differences in caloric content between the two categories. This a priori balancing was confirmed by a Mann–Whitney *U* test, which showed that the two categories did not differ significantly in energy density (MPF: 240 ± 188, HPF: 235 ± 131; W = 300.5, *p* = .823). The complete list of stimuli, divided into the two food categories (HPF and MPF) and reporting the caloric content (kcal/100 g) of each item, is provided in Table S1.

#### Sensorimotor and affective ratings

The assessment of food-related mental simulation was conceptually adapted from the Lancaster Sensorimotor Norms (Lynott et al., 2020).

For each word, participants rated the extent to which the concept elicited sensory experience across six modalities (interoception, haptics, audition, olfaction, gustation, vision). Specifically, they responded to the question: “*To what extent do you experience [WORD] through sensation inside your body/touch/hearing/smelling/tasting/seeing*?”, where “WORD” was replaced with each term of the list.

In contrast to Lynott et al. (2020), who assessed motor strength separately for individual effectors (e.g., hand/arm, foot/leg, torso, mouth/throat, head), the motor dimension in the present study was measured using a single, general question reflecting overall action engagement, consistent with the study’s focus on approach-related behavioral tendencies toward food. The question was phrased as: *“To what extent does the concept of [WORD] elicit the enactment of a behavioral action in you?”.* To control for potential confounding effects of lexical familiarity and semantic knowledge, participants also rated concept familiarity for each word, responding to the question: *“How familiar is the concept [WORD] to you?”*.

In addition to sensorimotor dimensions, liking and wanting were assessed for each food word, similarly to procedures adopted in previous word norm studies (Bradley & Lang, 1999; Repetto et al., 2022). For each item, presented individually on screen, participants responded to the following questions: *“How much do you like it?”* for liking and *“How much would you want to eat it right now?”* for wanting. All questions were originally administered in Italian and translated here for reporting purposes.

### Statistical analysis

Data were analyzed through R statistical software. Statistical significance was determined using an alpha level of 0.05.

As a first step, to investigate the effect of caloric content (CAL) and processing level (PROC) on mental simulation, linear mixed-effects models were conducted separately for each sensorimotor domain. In each model, the sensorimotor domain was included as the dependent variable, while PROC (HPF, MPF) and CAL, and their interaction were entered as fixed effects. Familiarity (FAM) and Body Mass Index (BMI) were included as covariates and participant ID and word were included as random effects to account for the repeated-measures structure of the data. CAL was mean-centered to facilitate the interpretation of main effects and interactions.

Models were fitted using the *lmer* function from the *lme4* package(Bates et al., 2015) according to the following specification: *mental_simulation* ~ PROC * CAL_c + FAM + BMI + (1 | participant) + (1 | word). PROC was dummy-coded with MPF as the reference level.

To further explore significant interactions between caloric content and processing level, simple slopes analyses were conducted. These analyses estimated the effect of CAL on mental simulation separately for processed and unprocessed foods. Slopes were derived from the model estimates using the emmeans package (Lenth, 2016), and significance was evaluated based on the 95% confidence intervals.

As a second step, given that we observed that the effect of caloric content and processing level was consistent across sensory modalities, a perceptual integration index was computed to capture global perceptual simulation strength. Specifically, a Minkowski distance–based index (order = 3) was calculated at the single-trial level by combining auditory, gustatory, haptic, interoceptive, olfactory, and visual ratings into a single perceptual score. In this metric, the highest-rated modality contributes most to the composite, while the remaining modalities add an attenuated contribution depending on how close their values are to the dominant one, thus providing a global index of perceptual simulation strength. Order = 3 has been identified as the optimal exponent for aggregating sensorimotor dimensions into a single composite (Lynott et al., 2020; To et al., 2011).

Using the Minkowski distance–based index, along with the motor simulation variable, we investigated the extent to which mental simulation predicted subjective evaluations of food. Linear mixed-effects models were conducted with wanting or liking as the dependent variable, and mental simulation (perceptual integration index or motor simulation) as the predictor of interest. Caloric content (CAL), processing level (PROC), familiarity (FAM), and BMI were entered as covariates. Participant ID and food item were included as random intercepts to account for the repeated-measures structure of the data. This approach allowed us to estimate the independent contribution of multisensory and motor simulations to subjective food evaluations, irrespective of the objectively defined properties of the foods. Models were fitted using the *lmer* function from the *lme4* package (Bates et al., 2015) according to the following specification: *wanting/liking* ~ mental simulation + PROC + CAL + FAM + BMI + (1 | participant) + (1 | word).

## Results

### Participants

The final sample consisted of 99 participants (37 males, 62 females), with a mean age of 34.49 years (SD = 14.38) and a mean BMI of 22.63 (SD = 3.56). Most participants were Italian (*n* = 97), with one Romanian and one Indian participant.

### Effects of Caloric Content and Processing Level on Mental Simulation of Foods

**Fig. 1:**
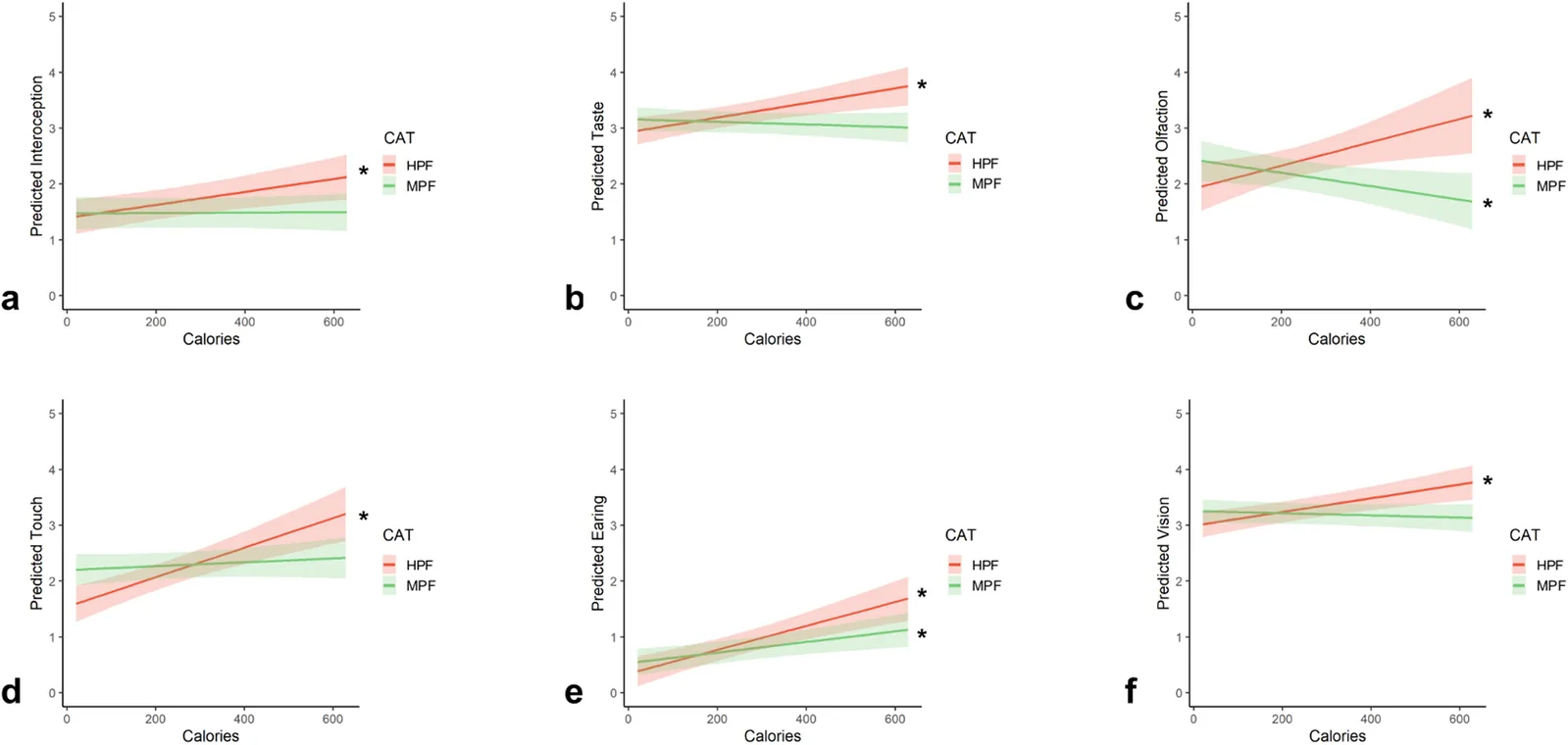
Mental simulation of foods across individual sensory modalities as a function of caloric content and processing category

**Fig. 2:**
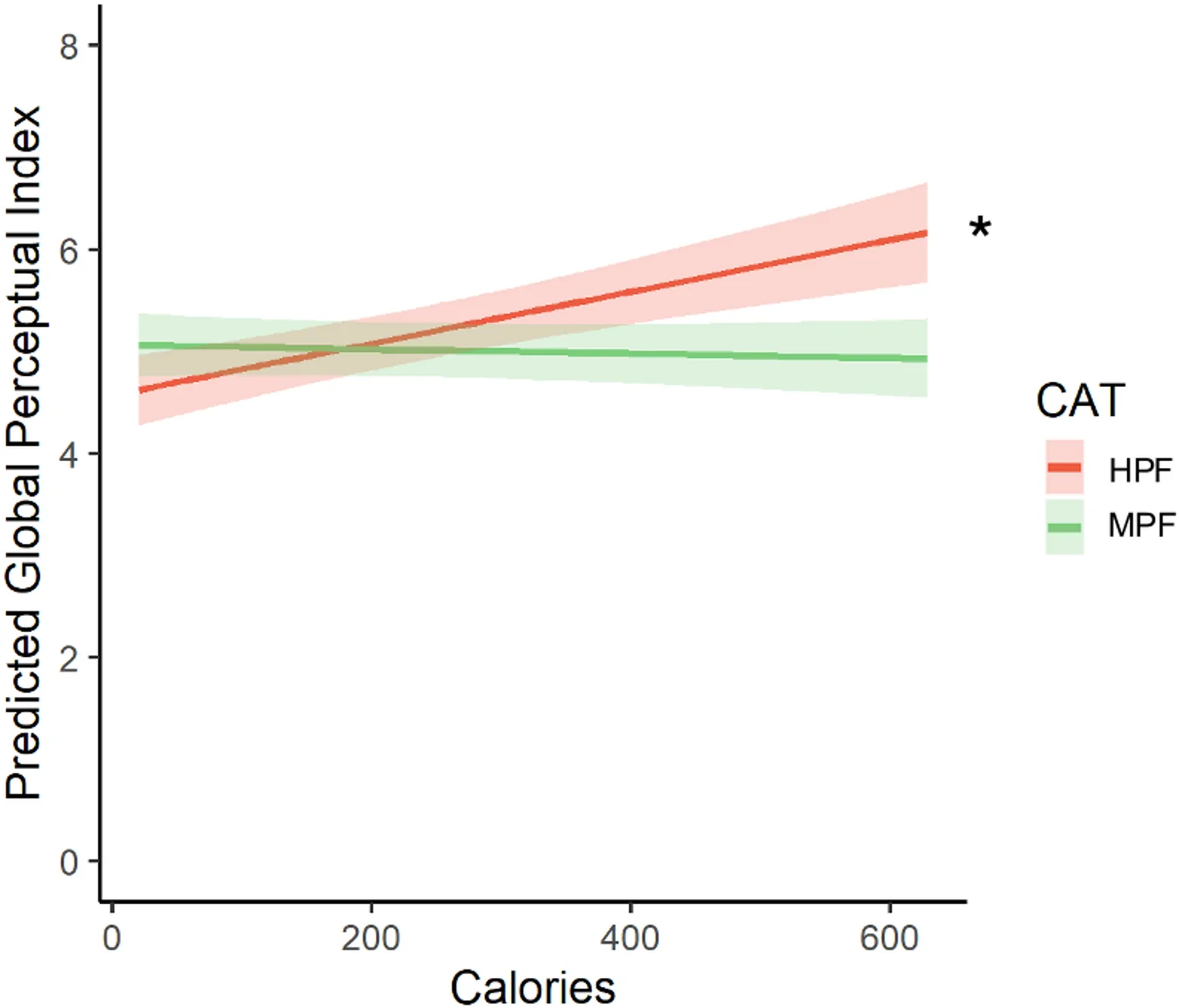
Mental simulation of foods as indexed by the global perceptual index (Minkowski3) as a function of caloric content and processing category

**Fig. 3:**
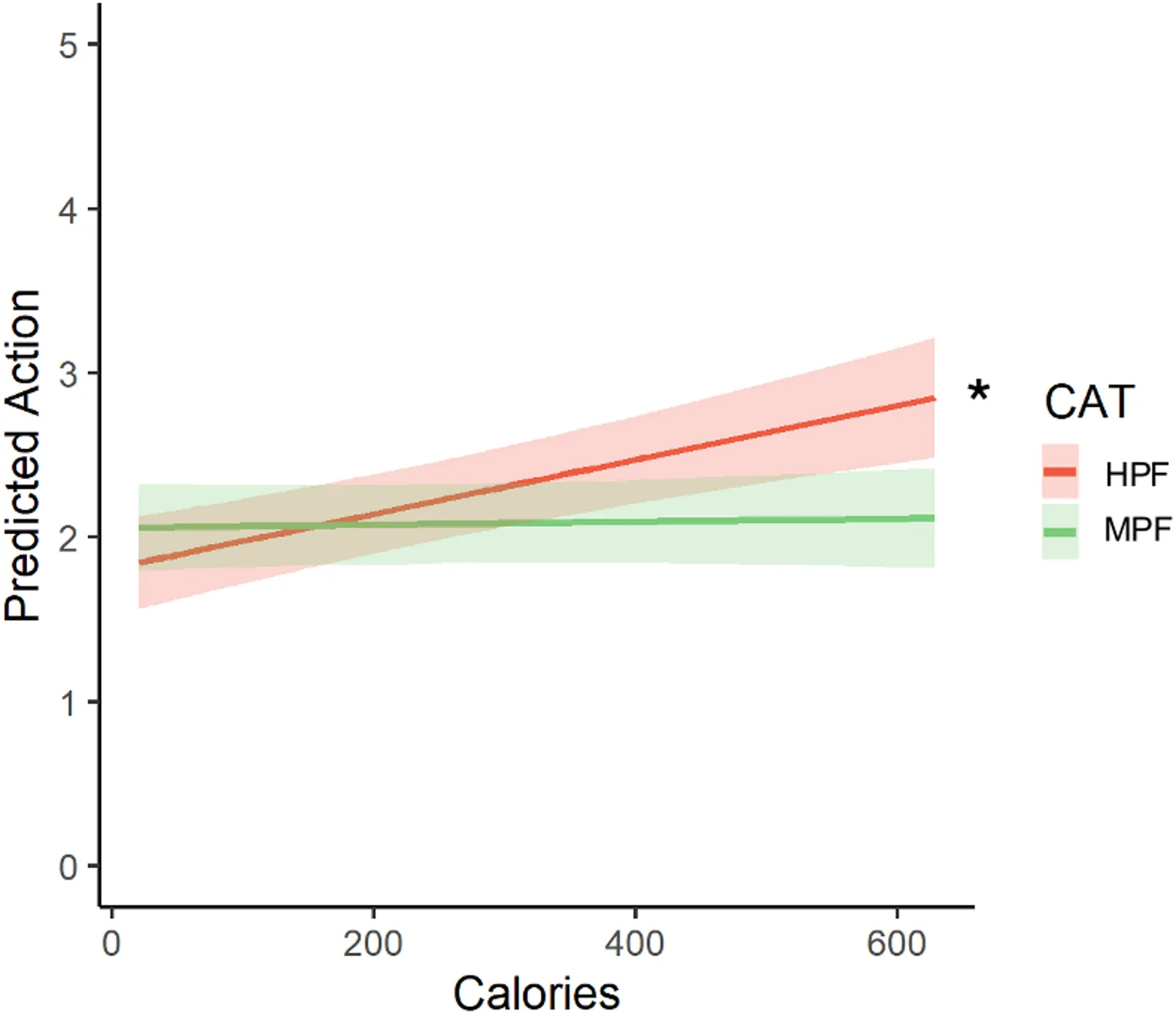
Mental simulation of foods across the action domain as a function of caloric content and processing category

The effects of caloric content and processing level on mental simulation across individual sensory modalities are illustrated in Fig. 1, while results for the global perceptual index and action domain are presented in Figs. 2 and 3, respectively.

#### Interoception

The effect of processing level was significant. Specifically, at mean caloric content, interoceptive responses were higher for HPF than MPF (β = 0.187, SE = 0.072, t = 2.62, *p* = .012).

The effect of caloric content was also significant (β = 0.00116, SE = 0.00040, t = 2.94, *p* = .005), indicating that higher-calorie foods elicited stronger interoceptive responses.

Importantly, the interaction between caloric content and processing level was significant (β = 0.00113, SE = 0.00048, t = 2.35, *p* = .023), showing that the positive effect of calories on interoceptive simulation was stronger for HPF compared with MPF. Consistently, follow-up analyses of the slopes revealed that while for HPF the relationship between caloric content and interoceptive simulation was positive and significant (slope = 0.001, 95% CI [0.0004, 0.0019]), for MPF the relationship was not significant (slope = 0.00003, 95% CI [-0.0005, 0.0006]).

#### Taste

The effect of caloric content was significant (β = 0.0013, SE = 0.0004, t = 3.49, *p* = .001), indicating that higher-calorie foods elicited stronger taste responses. Importantly, the interaction between caloric content and processing level was significant (β = 0.0015, SE = 0.0005, t = 3.37, *p* = .002), showing that the positive effect of calories on taste was stronger for HPF compared with MPF. Consistently, follow-up analyses of the slopes revealed that while for HPF the relationship between caloric content and gustatory simulation was positive and significant (slope = 0.001, 95% CI [0.0004, 0.0021]), for MPF the relationship was not significant (slope=-0.0002, 95% CI [-0.0008, 0.0003]).

#### Olfaction

The effect of caloric content was significant (β = 0.002, SE = 0.0008, t = 2.60, *p* = .012), indicating that higher-calorie foods elicited stronger olfactory responses. Importantly, the interaction between caloric content and processing level was significant (β = 0.003, SE = 0.0009, t = 3.35, *p* = .002). Specifically, follow-up slope analyses showed that for HPF, caloric content was positively associated with olfactory simulation (slope = 0.002, 95% CI [0.0005, 0.0037]), whereas for MPF, the relationship was negative (slope = -0.001, 95% CI [-0.0023, -0.0001]).

#### Touch

The effect of caloric content was significant (β = 0.003, SE = 0.0005, t = 4.86, *p* < .001), indicating that higher-calorie foods elicited stronger tactile responses. Importantly, the interaction between caloric content and processing level was significant (β = 0.0023, SE = 0.0007, t = 3.46, *p* = .001), showing that the positive effect of calories on touch was stronger for HPF compared with MPF. Consistently, follow-up analyses of the slopes revealed that while for HPF the relationship between caloric content and tactile simulation was positive and significant (slope = 0.003, 95% CI [0.002, 0.004]), for MPF the relationship was not significant (slope=-0.0004, 95% CI [-0.0004, 0.0011]).

#### Hearing

The effect of caloric content was significant (β = 0.002, SE = 0.0004, t = 4.83, *p* < .001), indicating that higher-calorie foods elicited stronger auditory responses. Importantly, the interaction between caloric content and processing level was significant (β = 0.0012, SE = 0.0005, t = 2.20, *p* = .033), showing that the positive effect of calories on hearing was stronger for HPF, compared with MPF. Follow-up analyses, however, revealed that caloric content was positively and significantly associated with auditory simulation for both HPF (slope = 0.0021, 95% CI [0.0013, 0.0030]) and MPF (slope = 0.0010, 95% CI [0.0003, 0.0016]).

#### Vision

The effect of caloric content was significant (β = 0.001, SE = 0.0003, t = 3.97, *p* < .001), indicating that higher-calorie foods elicited stronger visual responses. Importantly, the interaction between caloric content and processing level was significant (β = 0.0014, SE = 0.0004, t = 3.77, *p* < .001), showing that the positive effect of calories on vision was stronger for HPF compared with MPF. Consistently, follow-up analyses of the slopes revealed that while for HPF the relationship between caloric content and visual simulation was positive and significant (slope = 0.0012, 95% CI [0.0006, 0.0018]), for MPF the relationship was not significant (slope=-0.0002, 95% CI [-0.0006, 0.0002]).

#### Global perceptual index

The effect of caloric content was significant (β = 0.003, SE = 0.0005, t = 4.73, *p* < .001), indicating that higher-calorie foods elicited stronger perceptual responses. Importantly, the interaction between caloric content and processing level was significant (β = 0.0027, SE = 0.0006, t = 4.21, *p* < .001), showing that the positive effect of calories on perceptual simulation was stronger for HPF compared with MPF. Consistently, follow-up analyses of the slopes revealed that while for HPF the relationship between caloric content and global perceptual simulation was positive and significant (slope = 0.0026, 95% CI [0.0015, 0.0036]), for MPF the relationship was not significant (slope=-0.0002, 95% CI [-0.0009, 0.0005]).

#### Action

The effect of caloric content was significant (β = 0.002, SE = 0.0003, t = 4.68, *p* < .001), indicating that higher-calorie foods elicited stronger motor responses. Importantly, the interaction between caloric content and processing level was significant (β = 0.0016, SE = 0.0004, t = 3.62, *p* < .001), showing that the positive effect of calories on action was stronger for HPF compared with MPF. Consistently, follow-up analyses of the slopes revealed that while for HPF the relationship between caloric content and action simulation was positive and significant (slope = 0.0017, 95% CI [0.0009, 0.0023]), for MPF the relationship was not significant (slope = 0.0001, 95% CI [-0.0004, 0.0006]).

### Effects of mental simulation on wanting and liking for foods

#### Global perceptual index

**Fig. 4:**
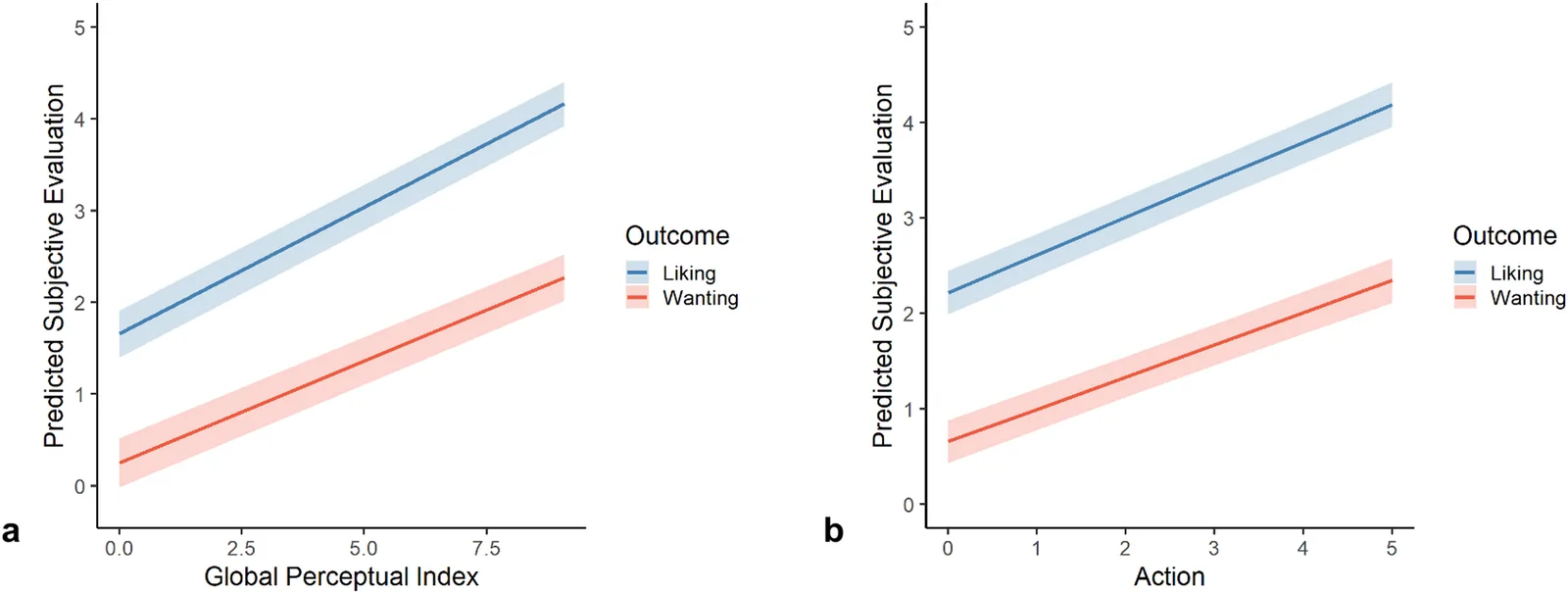
Effect of mental simulation on wanting and liking for foods. **a** Global perceptual index (Minkowski3); **b** Action domain

The effect of global perceptual simulation was significant for both wanting (β = 0.22, SE = 0.014, t = 15.53, *p* < .001) and liking (β = 0.28, SE = 0.013, t = 20.75, *p* < .001), indicating that higher levels of perceptual simulation were associated with greater wanting and liking, independently of objectively defined food characteristics (Fig. 4a).

#### Action

The effect of action was significant for both wanting (β = 0.34, SE = 0.017, t = 19.87, *p* < .001) and liking (β = 0.39, SE = 0.016, t = 24.96, *p* < .001), indicating that higher levels of motor simulation were associated with greater wanting and liking, independently of objectively defined food characteristics (Fig. 4b).

## Discussion

This study extends previous work by showing that the sensorimotor simulation of food concepts is shaped by objectively defined food properties, specifically by the interaction between caloric content and degree of industrial processing. Responses were analyzed at the level of individual participants and individual foods, preserving inter-individual variability that is lost when ratings are averaged. By focusing on objectively defined food properties rather than subjective healthiness and distinguishing between wanting and liking, our approach provides a richer understanding of the factors shaping food evaluation.

Notably, our results highlight a key interaction between caloric content and degree of processing in shaping sensorimotor simulation. This interaction emerged consistently across all sensory modalities, as well as in the global perceptual index and action-related ratings, indicating that these two properties jointly modulate the strength of sensory simulation and action engagement. While previous research has primarily contrasted foods based on perceived healthiness or caloric density (Muñoz-Vilches et al., 2020; Papies, 2013; Speed et al., 2023; Yang et al., 2021), our findings suggest that embodied food representations are more specifically modulated by the combination of caloric content and industrial processing. Importantly, although these dimensions may overlap at a subjective level (e.g. a food considered attractive being implicitly considered highly caloric and processed) (Alais et al., 2024; Foroni et al., 2013; Lemos et al., 2022), the present results indicate that processing constitutes a distinct and relevant variable that should be explicitly considered along with caloric content in experimental studies using food stimuli. Importantly, industrial processing should not be interpreted as a proxy for a single sensory property, but rather as indexing a broader constellation of features often associated with highly processed foods, including enhanced palatability, sensory complexity, and learned reward value (Gearhardt & DiFeliceantonio, 2023). Moreover, the results show that food concepts, particularly highly processed and high-calorie ones, engage a broad range of sensory modalities. Specifically, these stimuli elicited gustatory, visual, auditory, tactile, olfactory, and interoceptive dimensions. This pattern is consistent with the intrinsic properties of UPFs, which are characterized by enhanced sensory complexity, as well as bodily and interoceptive sensations that may accompany the anticipation or recollection of food consumption (Ghosh & Muley, 2025; Juul et al., 2025). From a grounded cognition perspective, such multisensory richness translates into stronger sensorimotor simulations, as similarly reported previously in a study using subjective healthiness ratings (Speed et al., 2023).

Critically, mental simulation was found to predict wanting and liking, as reflected in the global perceptual index and in action-related ratings: foods that elicited stronger simulations were perceived as more desirable and more pleasant. This finding is consistent with the grounded cognition theory of desire (Papies et al., 2017, 2020), according to which vivid, multimodal simulations of food cues increase craving by reactivating the reward associated with previous consumption. Notably, wanting and liking are conceptually related but partially dissociable components of food reward, relying on overlapping yet distinct neural systems (Berridge et al., 2009). In our data, simulation was positively associated with both wanting and liking, raising the theoretical possibility that it operates upstream of this dissociation, enriching the anticipatory representation of the food in a way that may feed into both motivational and hedonic processes rather than selectively driving one. Overall, these findings suggest that simulation processes not only reflect stored sensory experiences but may contribute to shaping food-related motivation and evaluation.

These results also suggest a possible cognitive mechanism linking food representation with the literature on UPFs consumption. It is well established that UPFs promote increased *ad libitum* energy intake and weight gain, even under controlled conditions, suggesting a facilitation of consumption beyond metabolic needs (Hall et al., 2019; Lemos et al., 2022). Moreover, habitual consumption of UPFs has been associated with a higher risk of obesity, metabolic disorders, and dysregulated eating patterns, including loss of control and addiction-like responses (Gearhardt & DiFeliceantonio, 2023; Gearhardt & Schulte, 2021; LaFata et al., 2024; Wiss & LaFata, 2024).

The present findings suggest that the same properties that facilitate consumption, such as high sensory intensity and hyper-palatability, also amplify mental simulation. These properties likely enhance the richness and strength of sensorimotor representations, which may involve a more extensive reactivation of multimodal experiences associated with prior consumption (L. W. Barsalou, 2008). In this sense, highly caloric and UPFs are not only easier to consume, but also more strongly represented at a sensorimotor level, a pattern that may be accompanied by enhanced reward-related signals and increased wanting. This interpretation provides a potential cognitive mechanism linking the physical properties of UPFs to their well-documented effects on overeating, offering a new perspective for understanding dysregulated eating patterns in clinical populations, such as in binge-eating disorder and bulimia nervosa. Indeed, UPFs constitute the majority of foods consumed during binge-eating episodes (Ayton et al., 2021; Worth et al., 2026), suggesting that they may contribute not only to general overconsumption but also to the maintenance of full-syndrome eating disorders. Given that the present study found high-caloric and ultra-processed foods to be jointly more effective at eliciting rich sensorimotor simulations, this tendency could be further amplified in clinical populations already characterized by heightened reward and interoceptive reactivity to food cues (Bronleigh et al., 2022; Celeghin et al., 2023), which could plausibly intensify craving and contribute to loss of control.

These findings have relevant implications for interventions aimed at promoting healthier eating behaviors. Current approaches predominantly focus on modifying the nutritional content of UPFs or increasing awareness (Capozzi et al., 2021; Monteiro et al., 2024; Robinson et al., 2024); however, the present results suggest that targeting the sensorimotor properties of food may be equally critical. If mental simulation contributes to food desirability, then enhancing the sensory richness, texture variability, and overall perceptual complexity of minimally processed foods may increase their capacity to elicit stronger simulations thereby improving their attractiveness and desirability. In this framework, minimally processed foods could be strategically reformulated or presented in ways that maximize their multisensory appeal, thereby narrowing the gap in desirability with UPFs. Such an approach may help shift food choices toward healthier and more sustainable options by leveraging the same mechanisms that currently drive preference for UPFs, rather than opposing them.

Nevertheless, these implications should be interpreted with caution. Although the present findings support a link between sensorimotor simulation and food-related motivation, the study relied exclusively on linguistic stimuli and self-reported measures. Therefore, it does not directly test whether stronger sensorimotor simulations translate into actual food choices or consumption behaviour. Future studies using more ecologically valid paradigms, including behavioural measures of food selection and intake, will be important to determine whether the mechanisms identified here generalise beyond conceptual processing.

Finally, some limitations must be acknowledged. To begin with, participants were recruited through online advertisements and snowball sampling, and the questionnaire was administered in Italian. This limits respectively the representativeness of the sample and the generalizability of findings beyond the Italian-speaking population. Another limitation is that the wide age range of the sample may have introduced variability in food ratings. Moreover, although the gender distribution was relatively balanced, the sample included a higher proportion of female participants. In addition, because the effect of food properties on simulation emerged as an interaction rather than as independent effects, a standard mediation model could not be appropriately estimated, thereby preventing us from formally establishing the association between objectively defined food properties, simulation, and food reward. Finally, food concepts were assessed using linguistic stimuli presented outside of an ecological context. Although this approach is consistent with the grounded cognition framework and the use of sensorimotor norms, real-world food choices typically involve direct multisensory experiences (e.g., visual, olfactory, gustatory, and tactile cues). Therefore, our findings do not fully capture the complexity of food evaluation and decision-making in everyday contexts.

### Future directions

Building on the limitations outlined above, future research should examine whether sensorimotor simulation patterns differ across age groups and gender in healthy individuals, as well as in clinical populations. In addition, the model used in the present study assumes relatively homogeneous responses across participants; however, individual differences such as dietary patterns, emotional eating tendencies, or hunger level are likely to modulate the strength of sensorimotor simulation to food cues, similarly to what has been reported for BMI in relation to neural responses to food cues (Chen et al., 2016). Moreover, future studies should directly test whether individuals with eating disorders characterized by loss of control and binge-eating episodes show a stronger sensorimotor simulation response to highly processed, high-calorie foods. In this respect, research on eating disorders should begin to explicitly consider UPFs as a distinct dimension of food stimuli, given their potential relevance for understanding the mechanisms underlying disordered eating. Finally, integrating more ecologically valid paradigms (e.g., real-life food exposure, immersive virtual environments) would help clarify how the proposed mechanisms operate in naturalistic settings and strengthen the translational relevance of the findings.

## Conclusion

In conclusion, the present study shows that sensorimotor simulation of food concepts varies systematically with objectively defined food properties, particularly the interaction between caloric content and degree of industrial processing. Simulation was also positively associated with wanting and liking, pointing to a possible cognitive mechanism through which highly processed, energy-dense foods may acquire increased motivational salience. These results extend current models of food evaluation by highlighting the role of embodied processes and suggest that considering the sensorimotor features of food may be relevant for understanding and modifying eating behavior.

## Supplementary Information

Below is the link to the electronic supplementary material.

Supplementary Material 1.

## Data Availability

Data, analysis scripts, and survey materials described in the manuscript will be made available upon request.
