# Supplementary material for: Caloric content and industrial processing modulate the mental simulation of food concepts
Source: Psychol Res. 2026 Sep 11;90(5):166. doi: 10.1007/s00426-026-02379-2 (PMC13569607; doi:10.1007/s00426-026-02379-2)
Supplement: Supplementary file 1 [file 426_2026_2379_MOESM1_ESM.pdf]

Appendix A - Supplementary Material

Title: Caloric Content and Industrial Processing Modulate the Mental Simulation of Food Concepts

Journal: Psychological Research

Authors: Sofia Gentili, Enrico Collantoni, Valentina Meregalli

Affiliation: Department of Neuroscience, University of Padua, Padova, Italy

Corresponding author: Valentina Meregalli: valentina.meregalli@unipd.it

Table A.1. *Stimuli words included in this study and their associated caloric content (kcal/100 g), translated from Italian into English.*

**HPF (kcal/100)**	**MPF (kcal/100)**
Chocolate (531)	Walnuts (589)
Cookies (426)	Pistachios (629)
Popcorn (408)	Almonds (628)
Croissant (403)	Cotton candy (394)
Candies (344)	Oatmeal (367)
Sausage (324)	Rice (367)
Cheeseburger (300)	Coconut (358)
Ice cream (291)	Barley (346)
Mortadella (288)	Pasta (341)
French fries(283)	Honey (304)
Wiener (259)	Dates (270)
Pizza (255)	Avocado (238)
Mozzarella (253)	Steak (187)
Chicken nuggets (242)	Salmon (185)
Jam (227)	Eggs (150)
Ricotta cheese (157)	Chicken (129)
Bresaola (152)	Artichokes (85)
Smoked salmon (147)	Banana (76)
Popsicle (137)	Shrimp (71)
Cottage cheese (115)	Potatoes (68)
Chickpeas (111)	Grapes (64)

Peas (68)	Raspberries (49)
Yogurt (66)	Orange (45)
Canned syrup fruit (56)	Spinach (41)
Tomato sauce (21)	Pumpkin (29)
